# Personality characteristics of empathy profiles – practical implications for education of medicine students

**DOI:** 10.1186/s12909-022-03432-5

**Published:** 2022-05-16

**Authors:** Barbara Bętkowska-Korpała, Anna Pastuszak-Draxler, Katarzyna Olszewska-Turek, Karolina Sikora-Zych, Roksana Epa, Anna Starowicz-Filip

**Affiliations:** 1 Department of Medical Psychology, Jagiellonian University Medical College, Jakubowskiego 2, 30-688 Cracow, Poland; 2 Department of Clinical Psychology, University Hospital in Cracow, Jakubowskiego 2, 30-688 Cracow, Poland; 3 Department of Psychiatry, University Hospital in Cracow, Kopernika 21a, 31-501 Cracow, Poland

**Keywords:** Profiles, Empathy, Personality, Big five, FFM, NEO-PI-R, Medical students

## Abstract

**Background:**

Empathy plays the key role in the doctor – patient relationship. The research of empathy determinants plays an important role in formulating practical guidelines for the education of medical students. The aim of this study was to analyse personality characteristics of empathy profiles among students of medicine, with consideration of chief personality factors and their subdimensions according to the FFM model.

**Methods:**

During workshops in Clinical Psychological Skills, 153 students (M = 57, F = 96; mean age 23 years) analysed their psychological functioning styles by examining their personality profiles and empathy indicators. Empathic Sensitiveness Scale (ESS) and Personality Inventory (NEO-PI-R) were applied for this purpose. The analyses of empathy indicators were presented by means of cluster analysis. Variance analysis with post hoc Tukey-b test was performed for differences between clusters and to differentiate between personality factors and their components in empathy clusters. This study was approved by the Jagiellonian University Bioethics Committee (approval number: 1072.6120.175.2018 date: 28.06.2018).

**Results:**

The first cluster included students who presented high empathetic concern for others, understood their perspective and needs characterised by medium level of Neuroticism, high levels of other dimensions The second group included students who could understand others very well, yet with lower tendency to react emotionally to suffering, characterised by medium level of Neuroticism, Extraversion, Openness, high Conscientiousness and low Agreeableness. The third cluster included students who react strongly to painful and unpleasant reactions of others, characterised by high Neuroticism and Agreeableness, low Extraversion.

**Conclusions:**

Each empathy profile is manifested in relations with patients in a specific way. Medical education in empathy holds great potential to reduce anxiety, stress, and burnout associated with the medical profession. Discussion of individual results with students, gives an opportunity to talk about how their empathy and personality characteristics may influence their everyday medical practice.

**Supplementary Information:**

The online version contains supplementary material available at 10.1186/s12909-022-03432-5.

## Introduction

The classic definition of empathy, proposed by Mark Davis [1, 2], emphasizes the ability to cognitively understand another’s perspective, as well as the emotional ability to recognize and respond to their emotional experiences. According to the author, this is the only way to, adequately interpret the influence of empathy level on individual’s behaviour. Davis [1, 2] highlights that both, cognitive aspect and emotional reactivity may influence reactions and behaviour in relations with others. Analysis of both aspects of empathy – cognitive and emotional one, enables further assessment of their influence on individual functioning in interactions with others. It is worth mentioning that a profile of empathy comprising both affective and cognitive components, can be described by three dimensions of empathy: Empathetic Concern (EC), Personal Distress (PD) and Perspective Taking (PT), each of them on a continuum from low to high.

The role of empathy in clinical practice is widely emphasized in literature. Empathy of medical doctors enhances positive relations with their patients [3, 4] and patients’ engagement in the treatment process in patient-centred care [5]. Results of one of the most recent studies [6] suggest that some aspects of empathy may influence students’ competences in communicating unfavourable prognoses to patients (Breaking Bad News).

Further studies confirm dependences between Davis’ empathy dimensions and personality factors in the Five-Factor Model (FFM or Big Five). Big Five, a broadly recognised personality model [7–9], proposes five chief dimensions of personality: Neuroticism (N), Extraversion (E), Openness (O), Agreeableness (A) and Conscientiousness (C). Each dimension comprises six subfactors, which allows for detailed analysis of basic tendencies and personality profiles.

Numerous authors have analysed an interesting phenomenon of dependence between personality traits and levels of empathy in medicine students and medical doctors [9–14]. Presented results confirm an unanimous relation between the level of Neuroticism and Personal Distress [11, 12]. Moreover, all components of Neuroticism have positive correlation with empathy [13]. Relation between Neuroticism and Perspective Taking are not that explicit. In a group of Chinese students, a positive correlation between Neuroticism and Perspective Taking has been observed [11]. A research carried out on a group of Polish students, showed negative correlations between Perspective Taking and three components of Neuroticism: Hostility, Impulsiveness and Vulnerability.

Another dimension of FFM, Extraversion, correlates positively with Empathetic Concern and Perspective Taking, and negatively with Personal Distress [11–13]. Extravert people, who are more focused on external world rather than emotions, and who seek contact with others [10] – are less susceptible to personal distress, as they are less concentrated on their “self”. In a study on communication between a general practitioner and a simulated patient, Extraversion and Emotion Recognition Ability, turned to be the most important factors that influenced positive assessment of the relation by patients [15]. The dimension of Openness and its subcomponents are in a positive correlation with Empathetic Concern and Perspective Taking [11, 13]. High levels of Openness, related to high flexibility in thinking and acting, and readiness to introduce changes, seem to be accompanied by empathetic approach to others [16].

Agreeableness has a strong positive correlation with all three dimensions of Empathetic Concern. Results of Polish research on students of medicine [13] are coherent with the results obtained from Chinese students of medicine [11] and with the results from Spain [12], where a positive correlation between Agreeableness, Empathetic Concern and Perspective Taking were observed. Available data show that people who are often caring about others, may feel inclined to resign from their own needs, in order to meet the needs of others (relations between Agreeableness and Empathetic Concern), which may frustrate them (relation between Agreeableness and Personal Distress). We therefore assume that people who belong to this group will also be more susceptible to personal distress when in contact with the suffering of others. At the same time, their specific approach – cognitive openness, will enable them to understand other person’s perspective (relations between Agreeableness and Perspective Taking).

The last dimension of the Big Five, Conscientiousness, is not directly related to any of the dimensions of empathetic sensitivity. Costa [10] state that Conscientiousness and empathy are two independent constructs with no correlations. Bętkowska-Korpała et al. [13], Song and Shi [11], Guilera et al.[12] indicate, that there is a correlation between Conscientiousness, Perspective Taking and Personal Distress.

Studies on interrelations between personality factors and empathy dimensions are valuable from theoretic perspective. Better understanding of this phenomenon is useful for development of psychosocial programs for students of medicine and medical doctors and may also be applied in further stages of professional development of medical doctors.

In 2018, we our research team carried out a study on a group of students of medicine, where we identified and described three profiles of empathy [17]. However, we were further interested in personality characteristics of students who comprised each profile, and wanted to know if there were any coherent personality characteristics, typical for each of the empathy profiles. In this research, our analyses were focused on describing empathy profiles of students of the fifth year of medical studies, with consideration of their five basic personality characteristics (Big Five) and their subfactors. Our analyses of previous studies, shows that examination of empathy profiles and their personality characteristics with consideration of subdimensions of the FFM model has not been done so far.

### Aim

The aim of this study was to analyse personality characteristics of empathy profiles among students of medicine, with consideration of chief personality factors and their subdimensions according to the FFM model.

## Material and method

The research group comprised of the students of medicine in their fifth year, (*n* = 153; M = 57, F = 96; the mean age of study participants was 23 years), recruited into the study in Workshops in Clinical Psychological Skills. Students who consented to participation in an anonymous study were filling in coded answer sheets. Informed and written consent was obtained from all individual participants included in the study.

### Measures

Two tools were applied in the research.

1. Personality Inventory (NEO-PI-R) [18, 19] describes the adopted taxonomy of 5 chief personality factors: Neuroticism, Extraversion, Openness, Agreeableness and Conscientiousness. Each of the main factors consists of six subfactors. The inventory consists of 240 items. Subjects use the Likert-type style (0 to 4) to indicate the truthfulness of a sentence in relation to themselves on an answer sheet. Raw results are calculated by means of keys and afterwards, taking into consideration the norms (for age and sex), the result is transferred to a sten value calculation sheet, Graph a personality profile. The Polish adaptation of this test is characterized by good psychometric indicators [8].
2. Empathic Sensitiveness Scale (ESS) [20] has been created on the basis of Davis’ theory and the Interpersonal Reactivity Index (IRI) tool [1, 2]. ESS adaptation comprises three indicators: Empathetic Concern (EC), Personal Distress (PD) and Perspective Taking (PT). The authors of the Polish adaptation did not include the sub-scale of Fantasy. This subscale seems to be the most problematics of all four identified parts of the IRI Questionnaire, which was pointed out by Davis [2], who wrote that is very difficult to unambiguously locate it within the theory and research model he proposes. It is also not without significance that even Davis often ignores the scale of Fantasy in his research [21] The EC indicator measures the tendency to express sympathetic affective reactions towards a person who experiences a difficult situation. The PD indicator shows the tendency to experience discomfort and unpleasant feelings in contact with a suffering person. Both above-mentioned indicators are affective. The third indicator—*PT* – a cognitive component of empathy, measures the ability to spontaneously take the perspective of other people and understand their point of view. ESS has norms for sten scores for the Polish population [20].

In the end of workshops, students receive form the leader, group feedback on general interpretation of results comprising the level of each factor, correlations between individual scales and explanation of their interrelations. Each student knows their results and can analyse in the group his/her empathy indicators and personality profile. The leader gives useful feedback and guidelines to the whole group. Students who are motivated to do a more detailed interpretation of their results can have individual consultations with the leader. In individual consultations, each student has the possibility to ask specific questions referring to his/her results and the way this knowledge about psychological functioning may be applied in practice in choosing specialisation, defining strengths and resources as well as areas for further attention and self-development. Students could learn about their personal resources and develop better understanding of their functioning in relation with patients.

### Statistical analysis

The Polish adaptation of tools applied in this study takes into account sex and age of subjects. Results were transferred to sten scores. Standard Ten Score (STEN) follow the normal distribution, with scale from 1 to 10, no negative values, statistically robust against measurement errors. It gives the possibility to compare individual results with the normalization group, and determine the range they fall into. In both tools the following sten ranges were applied: 1 and 2 – very low; 3 and 4 – low; 5 and 6 – average; 7 and 8 – high; 9 and 10 – very high [8]. The rule of rounding each result to the nearest whole number was applied in further discussion.

Empathy indicators (ESS) were analysed with cluster analysis with k-means clustering with constant interval distance sorting Euclidean algorithm. This method identified three clusters. Analysis of these clusters has already been presented [17], but it is attached in this article to enable its clearer understanding for the readers. In order to clarify further discussion, names of individual clusters indicate specific characteristic of each group (see Discussion). In the next step, variance analysis with post hoc Tukey-b test was applied for differences between clusters and to differentiate personality factors and their subscales between empathy clusters.

## Results

Three clusters of empathy were observed: the first cluster of 51 participants (34.46% of the group), the second cluster of 56 participants (37,84%), the third cluster of 41 participants (27.7%). Statistical parameters of clusters are presented in Table 1.

**Table 1:** Mean sten values and standard deviations in empathy clusters and variance analysis of differences between clusters

Empathy indicators ESS	Mean value			SD			*F*(2, 145)	*p*	Differences between clusters
	Cluster 1	Cluster 2	Cluster 3	S 1	S 2	S 3			
Empathetic Concern	7.71	3.93	5.63	1.32	1.44	1.64	89.76	< 0.001	1–2, 1–3, 2–3
Personal Distress	4.29	3.38	7.20	1.83	1.34	1.19	81.38	< 0.001	1–2, 1–3, 2–3
Perspective Taking	8.31	6.29	5.54	1.29	1.85	1.50	39.61	< 0.001	1–2, 1–3, 2–3

**Fig. 1:**
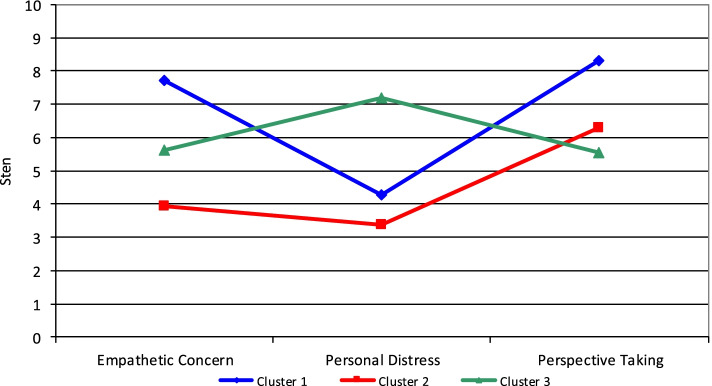
Mean sten values in clusters

Analysis of cluster 1., labelled „Strong Engagement”, shows high results in Empathetic Concern and Perspective Taking (mean sten scores 7.71 and 8.31) and low results of Personal Distress (mean sten score 4.29). Cluster 2 was labelled „Cognitive Processing”, because the highest scale in the group was Perspective Taking (mean sten score 6.29), whereas results for Empathetic Concern and Personal Distress were low (mean sten scores 3.93 and 3.38). Third cluster is characterised by a high level of Personal Distress (mean sten score 7.2), whereas results in the two other indicators: Empathetic Concern and Perspective Taking were medium (mean sten scores 5.63 and 5.54). This cluster was labelled „Personal Engagement”. Variance analysis of differences between clusters with post hoc Tukey-b test has shown differences between three clusters in all three empathy indicators (*p* < 0.001). Column „Differences between clusters “ presents couples of clusters with significant differences. Figure 1 presents a graph of mean sten values in clusters.

### Analysis of differences in personality characteristics between empathy clusters

In the next stage, variance analysis with post hoc Tukey-b test was performed to see if there were any differences in personality characteristics and their subdimensions between empathy clusters.

**Fig. 2:**
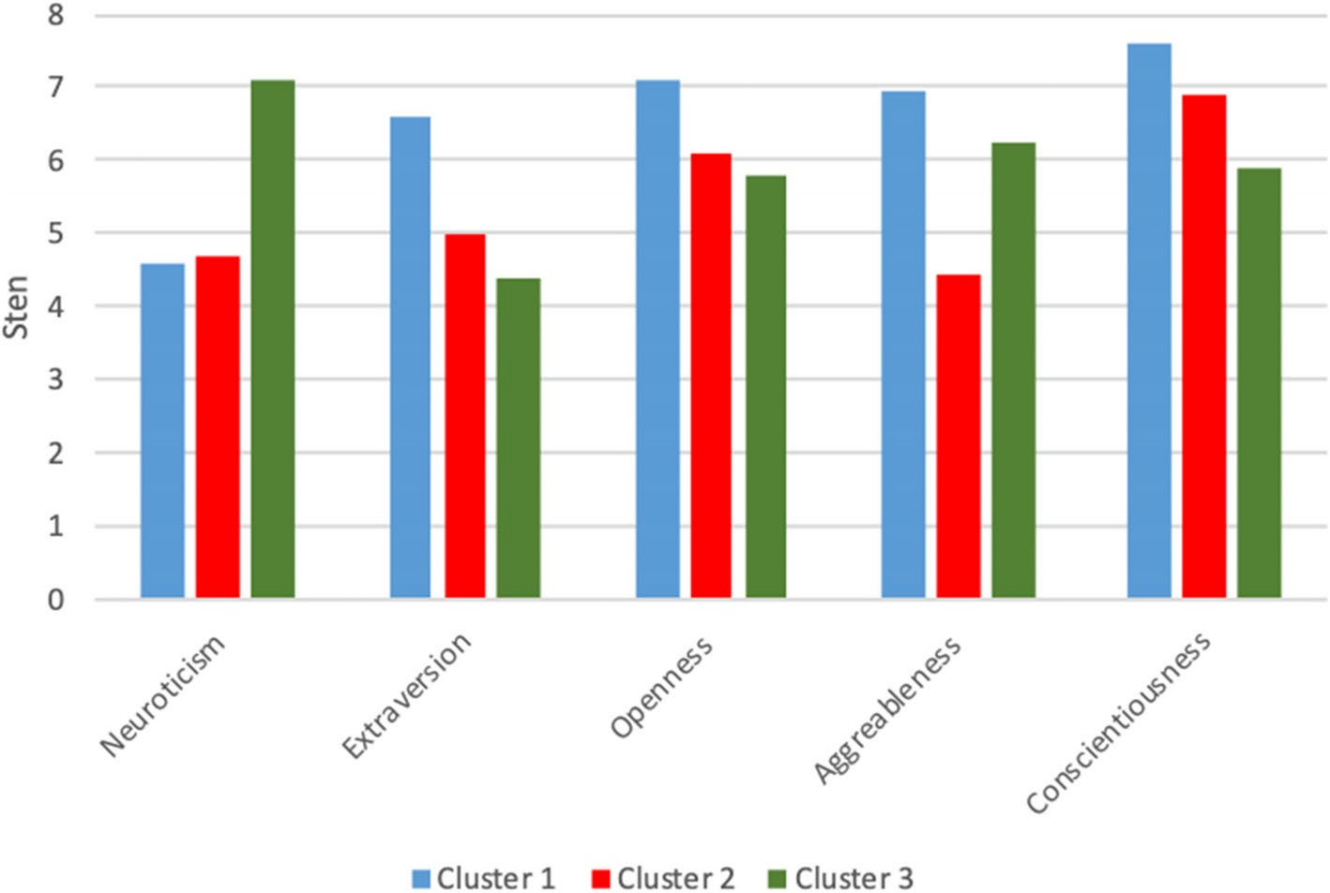
Mean sten values of the main personality dimensions in empathy clusters

Mean values and standard deviations in empathy clusters for each of the main FFM personality dimensions are presented in Table 2. Graphic presentation of sten values can be found in Fig. 2.

**Table 2:** Mean sten values and standard deviations of the main personality dimensions in empathy clusters

Personality factors	Mean value			SD			*F*(2, 145)	*p*	Differences between clusters
	Cluster 1	Cluster 2	Cluster 3	S 1	S 2	S 3			
N-Neuroticism	4.57	4.68	7.10	2.24	2.66	2.01	16.43	< 0.001	1–3, 2–3
E-Extraversion	6.57	4.98	4.39	2.05	2.41	1.92	12.96	< 0.001	1–2, 1–3
O-Openness	7.12	6.11	5.78	1.90	2.20	2.26	5.17	0.007	1–2, 1–3
A-Agreeableness	6.96	4.43	6.22	2.33	2.40	2.58	15.38	< 0.001	1–2, 2–3
C-Conscientiousness	7.59	6.91	5.88	2.00	2.27	2.64	6.35	0.002	1–3

Analysis has unveiled different personality characteristics in individual empathy clusters. As far as chief personality dimensions are concerned, Cluster 1 „Strong Engagement” and Cluster 2 „Cognitive Processing “ are characterised with medium level of Neuroticism (mean sten scores 4.57 and 4.68), which is not the case in Cluster 3 „Personal Engagement “ (mean sten score 7.10, *p* < 0.001). In Cluster 1, Extraversion was statistically higher (mean sten score 6.57, *p* < 0.001) than in Cluster 2 (mean sten score 4.98, *p* < 0.001) and Cluster 3, where Extraversion was low (mean sten score 4.39, *p* < 0.001). Analysis of Openness has shown that participants in Cluster 1 had higher results (mean sten score 7.12, *p* = 0.007) than participants in Clusters 2 and 3 (mean sten scores 6.11 and 5.78).

As far as Aggreableness is concerned, participants in Cluster 2 had lower results (mean sten score 4.43, *p* < 0.001), than participants in Clusters 1 and 3. (mean sten scores 6.96 and 6.22).

In the last dimension – Conscientiousness, there were significant differences between participants in Clusters 1 and 3 (mean sten scores 7.59 vs 5.88, *p* = 0.002).

Table 3 presents mean sten values and standard deviations of subdimensions of chief personality dimensions of the FFM in individual clusters. Variance analysis of differences between clusters has shown statistically significant differences. Further analyses by means of post hoc Tukey-b test, have shown differences between subdimensions of personality factors.

**Table 3:** Mean sten values and standard deviations of subdimensions

Personality factors	Mean value			SD						
	Cluster 1	Cluster 2	Cluster 3	S 1	S 2	S 3	*F*(2, 145)	*p*	Differences between clusters	
**Personality facets Neuroticism**										
N1-Anxiety	5.18	4.75	7.34	2.41	2.55	1.88	16.02	< 0.001	1–3, 2–3	
N2-Hostility, anger	4.47	4.98	5.73	2.53	2.15	2.27	3.36	0.037	1–3	
N3-Depression	4.94	4.64	7.02	2.50	2.65	2.25	12.13	< 0.001	1–3, 2–3	
N4-Self-consciousness	5.80	5.86	7.73	2.24	2.87	2.21	8.64	< 0.001	1–3, 2–3	
N5-Impulsiveness	5.88	6.18	6.68	2.66	2.54	2.05	1.22	0.300		
N6-Vulnerability to stress	3.47	4.14	6.66	2.13	2.39	1.82	26.99	< 0.001	1–3, 2–3	
**Personality facets Extraversion**										
E1-Kindness	7.69	4.95	5.59	1.93	2.40	2.56	20.22	< 0.001	1–2, 1–3	
E2-Gregariousness	5.92	5.23	5.10	2.27	2.42	1.66	2.00	0.139		
E3-Assertiveness	6.76	5.73	4.24	2.47	2.64	2.31	11.64	< 0.001	1–3, 2–3	
E4-Activity	6.45	5.71	5.02	2.32	2.46	2.34	4.12	0.018	1–3	
E5-Excitement seeking	4.76	4.61	4.66	2.30	2.20	1.85	0.07	0.929		
E6-Positive emotion	7.45	5.79	5.15	2.15	2.78	2.73	10.24	< 0.001	1–2, 1–3	
**Personality facets Openness to experience**										
O1-Imagination	6.41	5.93	6.41	2.01	2.48	2.38	0.77	0.463		
O2-Aesthetics	6.18	4.64	5.12	2.65	2.37	2.53	5.11	0.007	1–2	
O3-Emotionality	7.08	5.13	5.68	2.30	2.57	2.48	8.78	0 < .001	1–2, 1–3	
O4-Actions	6.39	6.16	4.59	2.16	2.46	1.94	8.68	< 0.001	1–3, 2–3	
O5-Ideas	7.25	7.23	6.17	2.24	2.34	1.92	3.52	0.032	1–3, 2–3	
O6-Values	7.20	7.82	7.22	2.54	2.51	2.42	1.06	0.350		
**Personality facets Agreeableness**										
A1-Trust in others	7.04	5.34	5.98	2.31	3.03	2.67	5.35	0.006	1–2	
A2-Straightforwardness	6.51	4.96	6.68	2.18	2.49	2.41	8.27	< 0.001	1–2, 2–3	
A3-Altruism	7.43	4.54	5.66	1.88	2.72	2.65	18.91	< 0.001	1–2, 1–3	
A4-Compliance	6.73	5.41	6.02	2.10	2.09	2.04	5.34	0.006	1–2	
A5-Modesty	5.86	4.41	6.27	2.75	2.29	2.18	8.18	< 0.001	1–2, 2–3	
A6-Tendermindedness	6.25	4.57	5.68	2.50	2.79	2.95	5.22	0.006	1–2	
**Personality facets Conscientiousness**										
C1-Competence	8.35	7.79	6.24	1.85	2.32	2.53	10.63	< 0.001	1–3, 2–3	
C2-Organizing	6.88	6.36	5.93	2.52	2.59	2.48	1.64	0.198		
C3-Dutifulness	7.63	6.70	6.51	1.99	2.11	2.05	4.14	0.018	1–2, 1–3	
C4-Achievement Striving	7.14	6.95	6.05	2.32	2.82	2.84	2.11	0.124		
C5-Self-Discipline	6.73	6.59	5.12	2.60	2.18	2.58	5.87	0.004	1–3, 2–3	
C6-Deliberation	7.20	6.45	6.46	2.03	2.35	2.18	1.91	0.152		

There were no differences between clusters in one subdimension od Neuroticism, namely Impulsiveness (N5). Participants in Cluster 3 „Personal Engagement “, presented statistically higher results (*p* < 0.001) than participants in Clusters 1 and 2 in the following subdimensions: Anxiety (N1: 7.34 vs 5.18 and 4.75), Depression (N3: 7.02 vs 4.94 and 4.64), (N4: 7.73 vs 5.8 and 5.86), (N6: 6.66 vs 3.47 and 4.14). Moreover, participants in Cluster 1 presented highest results in Angry Hostility, which were however on the mean level for the population (N2: 5.73 vs 4.47 and 4.98, *p* = 0.037). Graphic presentation of the Neuroticism subdimensions can be found below in Figure S1 (Additional file 1).

There were differences between clusters in four subdimensions of Extraversion. Cluster 1 „Strong Engagement “ is characterized with statistically higher results (*p* < 0.001) than the other two Clusters in: Kindness (E1: 7.69 vs 4.95 and 5.59) and Positive Emotions (E6: 7.45 vs 5.79 and 5.15). Further differences were observed between Cluster 1 and 3 in: Assertiveness (E3: 6.76 vs 4.24; *p* < 0.001) and Activity (E4: 6.45 vs 5.02; *p* = 0.018). There were differences in Assertiveness between Cluster 2 and 3 (E3: 5.73 vs 4.24; *p* < 0.001). Graphic presentation of the Extraversion subdimensions can be found below in Figure S2 (Additional file 1).

There were differences between Clusters in four subdimensions of Openness to Experience. Participants in Cluster 1 „Strong Engagement “ presented higher results in Emotionality, comparing to Cluster 2 and 3 (O3: 7.08 vs 5.13 and 5.68; *p* < 0.001), as well as in Aesthetics, comparing to Cluster 2 (O2: 6.18 vs 4.64 and 5.12; *p* = 0.007). Participants in Cluster 3, presented lower results in Actions and Ideas, than participants in two other Clusters (O4: 4.59 vs 6.39 and 6.16; *p* < 0.001, O5: 6.17 vs 7.25 and 7.23; *p* = 0.032). However, these results were on the average level for the population. Graphic presentation of the Openness subdimensions can be found below in Figure S3 (Additional file 1).

There were differences between clusters in all subdimensions of Agreeableness. Subdimensions of Trust in others, Compliance and Tendermindedness were higher in Cluster 1 than in Cluster 2 (A1: 7.04 vs 5.34; U4: 6.73 vs 5.41; A6: 6.25 vs 4.57; *p* = 0.006). Subdimensions of Straightforwardness and Modesty in Cluster 2 were lower than in Clusters 1 and 3 (A2: 4.96 vs 6.51 and 6.68, A5: 4.41 vs 5.86 and 6.27; *p* < 0.001). Subdimension of Altruism was the highest Cluster 1 (A3: 7.43 vs 4.54 and 5.66; *p* < 0.001). Graphic presentation of the Agreeableness subdimensions can be found below in Figure S4 (Additional file 1).

There were statistical differences between clusters in three subdimensions of Conscientiousness. The highest results were observed in Cluster 1. Subdimension of Competence and Self-Discipline in Cluster 3 were lowest (yet within mean sten values for population), comparing to Cluster 1 and 2 (C1: 6.24 vs 8.35 and 7.79; *p* < 0.001, C5: 5.12 vs 6.73 and 6.59; *p* = 0.004). Subdimension Dutifulness was the highest in Cluster 1 (C3: 7.63 vs 6.70 and 6.51; *p* = 0.018). There were no differences between sten values in the remaining three subdimensions of Conscientiousness. Graphic presentation of the Conscientiousness components can be found below in Figure S5 (Additional file 1).

**Fig. 3:**
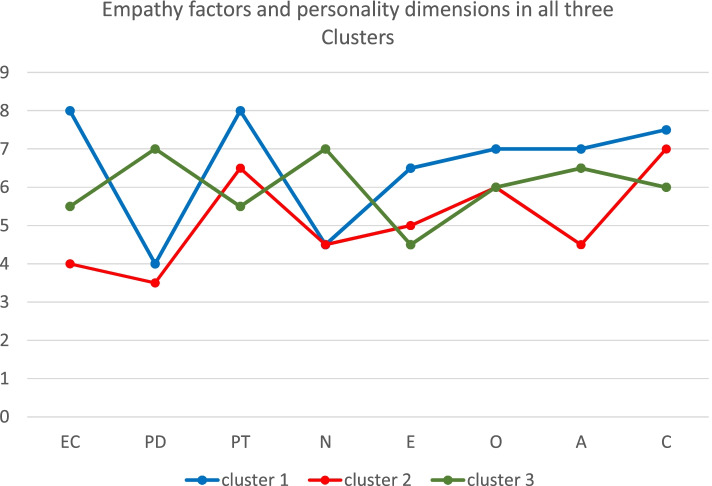
Empathy factors and personality dimensions in all three clusters

Figure 3 shows aggregate results of empathy factors and personality dimensions in all three clusters.

## Discussion

Three profiles of medical students’ empathy were described in this study. There were statistically important differences between these profiles in various indicators of empathy. Each profile is manifested in a specific way in relation to patients and has specific meaning for future functioning of medical doctors in their activities. These profiles were described in a detailed way in our previous studies [17], yet for the sake of this publications we present their labels and short descriptions in the Table 4 below.. Indicators in the first profile “Strong Engagement” show that participants in this category are characterised by strong empathetic concern for other people, they understand their perspective and needs, yet at the same time, they do not get disturbed when they participate in a situation that is emotionally difficult for other people. Empathy indicators in the second profile “Cognitive Processing” show that participants in this category can understand situation of other people but have lower tendency to react emotionally to their suffering. They have lower ability to engage emotionally and express care for others. The third profile “Personal Engagement” comprises students, who react strongly to situations that are difficult to other people. They present empathetic behaviours and understand perspective of others. Our study has shown, that there are statistically important differences between these profiles as far as FFM personality dimensions are concerned, which means that students belonging to each profile have different personality characteristics. The “Strong Engagement” profile is characterised by medium level of Neuroticism and its subdimensions, and high levels of other dimensions, as well as high or medium levels of their subdimensions. The only subdimension with low scores is, Vulnerability to stress (N6), which indicates an effective ability of coping with stress (Table 4). To conclude the analysis of this profile, we may say that these students have good emotional adaptation, are less susceptible to negative emotions and more ready to experience positive emotions. They seek interpersonal contacts and can cooperate. High level of Openness and Conscientiousness indicate that they can creatively engage in relations and activities. Therefore we can prognose, that in medically difficult situations, that stimulate empathetic engagement, they will get emotionally and cognitively engaged, at the same time relying on their self-protective mechanisms, being able to treat difficult situations as challenges. Results of this study are coherent with reports from other authors, who observed clear correlations between high levels of some personality dimensions described by Costa and McCrae [7, 22] and specific dimensions of empathy. For example, Mooradian and his colleagues [23], have observed that the level of Agreeableness has strong correlation with empathetic concern and medium correlation with cognitive empathy (perspective taking). In a transcultural study carried out on a sample of students from China, Germany, Spain and the USA, it was shown that Agreeableness is the most important personality dimension related to perspective taking and empathetic concern [24]. Similarly, Openness to experience is described as a dimension that correlates positively with perspective taking, and negatively with the tendency to experience personal distress [11, 25], which is coherent with our observations. In our previous article, we have indicated on a correlation between the level of Agreeableness, Openness to experience and Extraversion with Empathetic Concern and Perspective Taking, that was additionally correlated positively with the level of Conscientiousness [13].

**Table 4:** Characteristic of empathy dimensions and personality in each of the three clusters

Clusters	Characterstics of empathy dimensions and personality
Cluster 1: Strong engagement	Empathetic Concern (high), Perspective Taking (high) Personal Distress (low) Neuroticism (medium) and other personality dimensions (high). subdimension Vulnerability to stress (low)
Cluster 2: Cognitive processing	Perspective Taking (high), Empathetic Concern (low), Personal Distress (low) Neuroticism (medium), Extraversion (medium), Openness to experience (medium), Conscientiousness (high) Agreeableness (low)
Cluster 3: Personal engagement	Personal Distress (high), Empathetic Concern (high), Perspective Taking (medium) Neuroticism (high), Openness to experience (medium), Agreeableness (medium), Conscientiousness (medium), (high) (high), Extraversion especially Assertiveness (low)

The second profile “Cognitive Processing”, comparing to the other two profiles, is characterised by a medium level of Neuroticism, Extraversion, Openness to experience, high Conscientiousness and low Agreeableness – especially in subdimensions of Straightforwardness and Modesty (Table 4). Bearing in mind constellation of personality traits in students comprising this cluster, we may deduce that they are characterised by task-orientation, high ambitions and motivation for achievements, feel highly competent and important in the group [8]. Few research articles have been published so far, on the relation between Conscientiousness and cognitive empathy (the dimension of Perspective Taking). This correlation was observed by Bętkowska-Korpała with her colleagues [13] and Airagnes with his colleagues [26]. In the second of the above-mentioned articles, it has been noted that students of the fourth year of medical studies who are highly conscientious, extravert and not very neurotic, have better developed cognitive empathy, than their colleagues with other personality profiles [26].

The third profile, „Personal engagement”, is characterised by high Neuroticism in almost all its subdimensions and low Extraversion, especially Assertiveness (E3) (Table 4). Comparing to population norms, students in the third empathy profile, due to high neuroticism, have the tendency to experience negative emotions, are less effective in coping with stress, slower in taking actions and more self – critical. They also have the tendency to give way to others [8]. We may prognose, that in difficult clinical situations, students with such empathy profile, domination of personal engagement and abovementioned constellation of personality traits will bear high emotional costs, which increases the risk of professional burnout [27–29]. High level of neuroticism is often described in the context of a strong tendency to experience intense personal distress in contact with a suffering person [11, 23, 24, 30] It is generally recognised, that the two basic aspects of neuroticism are susceptibility to experiencing negative emotions and difficulty in regulating own emotional reactions [22]. Experience of empathetic personal distress is related to concentration on own discomfort and anxiety [31] and inability to effectively regulate these emotions [32]. Therefore both constructs are similar, especially if we consider a situation when such a person witnesses suffering of another person. It may explain an often described relation between the level of neuroticism and a high level of personal distress. The Table 4. below presents a general characteristic of empathy dimensions and personality in each of the three clusters.

### Practical implications of the study

Results of our study have significant practical application. They have a diagnostic meaning, as they allow us to identify personality and empathy resources and limitations (cognitive – emotional), that can be important in the career of future medicine doctors, also in the context of contact and relations with their patients.

Secondly, these results can form an important point of reference in further choosing of medical specialisation. Plaisant et al. [33], argue that medicine is so complex and diverse, that medical doctors, who specialise in different fields, need different personality characteristics. Taking into account various resources and limitations observed in subjects of the study in individual clusters, we may presume that subjects belonging to Cluster 1 „Strong Engagement” will most probably function well as specialists in numerous fields of medicine. On the other hand, fields of specialisation that require high concentration of patients’ emotions and understanding them, with little concentration on own difficult emotions are palliative medicine, oncology or chronic conditions in paediatrics. Liew and Azim (2021) [34] showed that medical students who stated a preference for future specialization that requires more communication with patients scored higher on the Empathetic Concerns and Perspective Taking subscales. Mullola et al. [35] focused on the question, whether personality traits moderate a relationship between a medical doctor’s well-being and his/her specialisation. It has turned out, that higher extraversion, openness to experience and agreeableness are related to higher well-being in medical doctors who have person-oriented specialisations. In our study, students from the „Strong Engagement” group (first profile), present this characteristics.

Subjects form the Cluster “Cognitive Processing” seem to have multiple cognitive, emotional and personality related resources that are necessary in doctors specialising in invasive and interventive medicine. Lower openness and lower agreeableness were beneficial for well-being of medical doctors, who chose technique-oriented specialisations (Mullola et al. [35].

High level of empathy and understanding of patients is an unquestionable advantage of subjects from the third Cluster “Personal Engagement”, yet concentration on own difficult emotions may make focusing on patients more difficult for these subjects. It is difficult to show a concrete specialisation in which these characteristics are specifically required. It seems that self-reflection of a doctor and his/ her openness to understanding themselves may protect them against professional burnout and mobilise to develop own resources which would be beneficial in each medical specialisation.

In the context of our research results, it needs to be remembered, that certain personality and emotional characteristics can be developed or even modified by means of various techniques of shaping empathy (*didactics, reflection exercises, simulations, virtual hangouts, technology-enhanced interventions) and become a resource* (Menezes et al.,2021) [36]. Leading techniques applied in education in the field of empathy, that positively influence its development in students of medicine, are those increasing communication skills, mindfulness, early clinical exposition, technology supported learning and humanistic aspects (Menezes et al.,2021) [36].

Workshops lead by our staff, that are described in section Methods can play similar role. Below, there is a short presentation of how subjects of our studies from three clusters may apply knowledge that they gain in clinical workshops run by our psychologist’s team to recognize their resources and potentially weak points in further clinical work.

Results of our study show that students of medicine may need different trainings in psychological skills.

Students from the „Strong Engagement “ group, who received high scores in subdimensions of Empathic Concern and Perspective Taking, may benefit from training in recognising what clinical situations/ what types of patients evoke their compassion and the wish to take care about them. High resources of empathic concern that can be found in this group, may be further developed in psychological trainings based on discussing feelings and thoughts in relation to individual patients.

For students from the group “Cognitive Processing”, it would be valuable to help them develop emotional sensitivity and openness to emotions – both, their own and others’. It would help them greatly in their everyday work with patients, who often express deep emotional needs towards them.

Students belonging to the group labelled as “Personal Engagement”, may be more susceptible to professional burnout than those from two other groups. David J. Prins [37] observed, that high levels of neuroticism and low extraversion correlate with higher risk of professional burnout. Song et al. [11] described how high neuroticism and low extraversion, agreeableness and conscientiousness are related to the levels of stress and depressive symptoms. This context reminds us of an interesting issue, namely, a link between certain personality traits and the tendency to experience unpleasant feelings in contact with suffering people, and susceptibility to depressive states. For the last twenty years, scientists interested in human psychological development, have observed correlations between high sensitivity to suffering of others, tendency to long term worrying and experiencing of guilt and susceptibility to depressive states [36–38]. In the interpersonal context, this means sensitivity to the emotional reactions of others and high discomfort. This kind of reactivity in relation to others often leads to emotional overburdening (that may lead to avoidance of contact and withdrawal, resignation form helping), loss of satisfaction from work and personal life.

Considering the abovementioned remarks, It seems that these students could benefit from training where they could observe their personal characteristics revealed in relation with people in need of help, and that would give them an opportunity to talk about their difficult experiences in a safe environment. It could help them develop higher sensitivity to their own emotions and emotions of others (colleagues and patients), which would be a preventive measure, protecting them against professional burnout and depressive states.

In our workshops, we encourage students to make predictions of how their personality related and empathy resources can be applied in various fields of medical specialisations. Our observations so far have shown us that predictions of students are coherent with predictions made by the staff of clinical psychologists, who lead our workshops. Quite often this gives the opportunity to analyse their own personality and empathy characteristics in order to choose their further professional development path.

### Limitations

Our study is not free from limitations. We applied only self-descriptive methods. Empathy was not measured externally in natural clinical situations. Personality testing by a self-descriptive tool always provides a subjective perspective of subjects. We may also consider as a limitation the fact, that the group of subjects comprises a relatively homogenous group (fifth year’s students) which limits extrapolation of results on all students of medicine.

## Conclusions

It is possible to divide students of medicine into three coherent groups, characterized by similar dimensions of empathy. These groups differ in characteristics and personality profiles according to the Big Five Model. Distinction of students with similar empathy characteristics and personality styles, gives an opportunity to better adjust and profile training techniques, as well as a choice of future medical specialization.

## Supplementary Information

**Additional file 1: Figure S1.** Mean sten values in individual clusters of the Neuroticism subdimensions. **Figure S2.** Mean sten values in individual clusters of the Extraversion subdimensions. **Figure S3.** Mean sten values in individual clusters of the Openness subdimensions. **Figure S4.** Mean sten values in individual clusters of the Agreeableness subdimensions. **Figure S5.** Mean sten values in individual clusters of the the Conscientiousness subdimensions.

## Data Availability

The datasets generated and analysed during the current study are available from the corresponding author upon request.

## Abbreviations

ESS: Empathic Sensitiveness Scale
NEO-PI-R: Personality Inventory
FFM: Five-Factor Model (or Big Five)
EC: Empathetic Concern
PD: Personal Distress
PT: Perspective Taking
N: Neuroticism
E: Extraversion
O: Openness
A: Agreeableness
C: Conscientiousness
IRI: Interpersonal Reactivity Index
N1: Anxiety
N2: Hostility, anger
N3: Depression
N4: Self-consciousness
N5: Impulsiveness
N6: Vulnerability to stress
E1: Kindness
E2: Gregariousness
E3: Assertiveness
E4: Activity
E5: Excitement seeking
E6: Positive emotion
O1: Imagination
O2: Aesthetics
O3: Emotionality
O4: Actions
O5: Ideas
O6: Values
A1: Trust in others
A2: Straightforwardness
A3: Altruism
A4: Compliance
A5: Modesty
A6: Tendermindedness
C1: Competence
C2: Organizing
C3: Dutifulness
C4: Achievement Striving
C5: Self-Discipline
C6: Deliberation
