# Supplementary figures and images for: Personality characteristics of empathy profiles – practical implications for education of medicine students
Source: BMC Med Educ. 2022 May 16;22:376. doi: 10.1186/s12909-022-03432-5 (PMC9112556; doi:10.1186/s12909-022-03432-5)

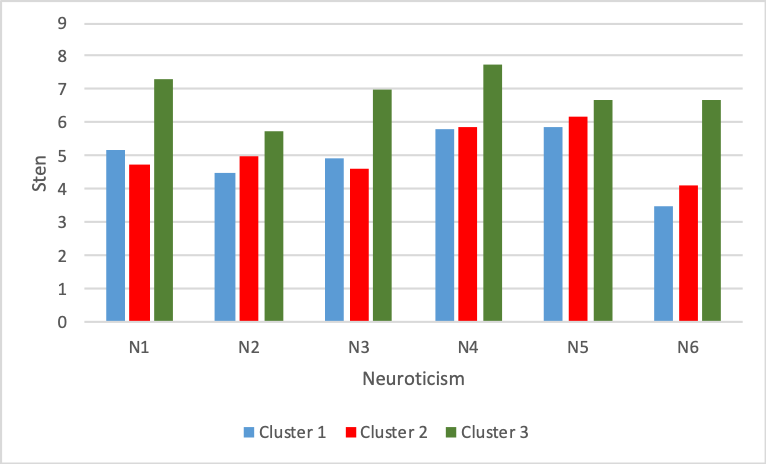


Figure S1. Mean sten values in individual clusters of the Neuroticism subdimensions.

Supplement: Supplementary file 1 — Additional file 1: Figure S1. Mean sten values in individual clusters of the Neuroticism subdimensions. Figure S2. Mean sten values in individual clusters of the Extraversion subdimensions. Figure S3. Mean sten values in individual clusters of the Openness subdimensions. Figure S4. Mean sten values in individual clusters of the Agreeableness subdimensions. Figure S5. Mean sten values in individual clusters of the the Conscientiousness subdimensions. [file 12909_2022_3432_MOESM1_ESM.zip › 1-Fig S1.docx]

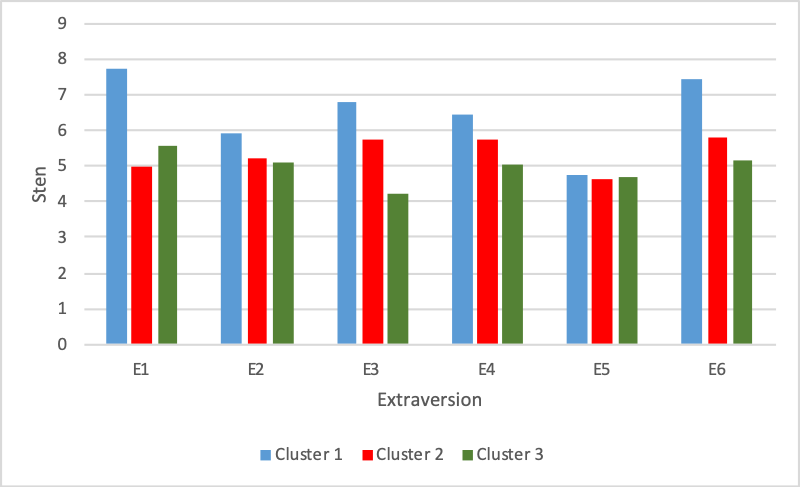


Figure S2. Mean sten values in individual clusters of the Extraversion subdimensions

Supplement: Supplementary file 1 — Additional file 1: Figure S1. Mean sten values in individual clusters of the Neuroticism subdimensions. Figure S2. Mean sten values in individual clusters of the Extraversion subdimensions. Figure S3. Mean sten values in individual clusters of the Openness subdimensions. Figure S4. Mean sten values in individual clusters of the Agreeableness subdimensions. Figure S5. Mean sten values in individual clusters of the the Conscientiousness subdimensions. [file 12909_2022_3432_MOESM1_ESM.zip › 1-Fig S2.docx]

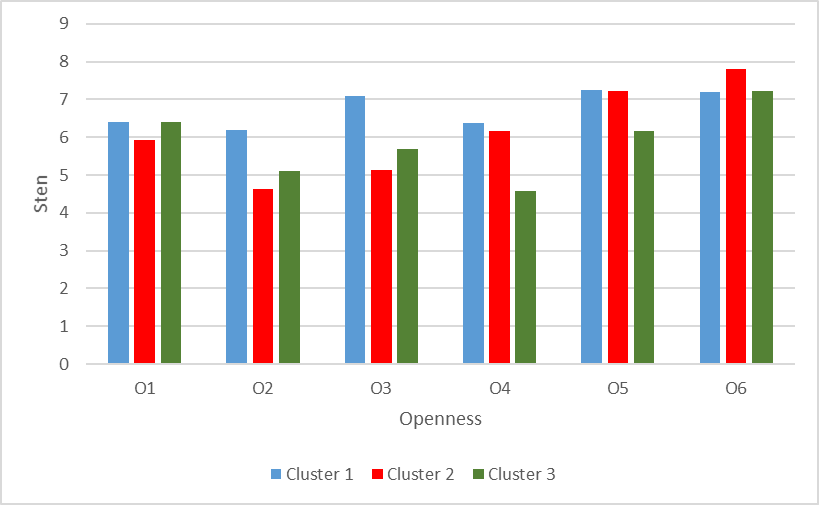


Figure S3. Mean sten values in individual clusters of the Openness subdimensions

Supplement: Supplementary file 1 — Additional file 1: Figure S1. Mean sten values in individual clusters of the Neuroticism subdimensions. Figure S2. Mean sten values in individual clusters of the Extraversion subdimensions. Figure S3. Mean sten values in individual clusters of the Openness subdimensions. Figure S4. Mean sten values in individual clusters of the Agreeableness subdimensions. Figure S5. Mean sten values in individual clusters of the the Conscientiousness subdimensions. [file 12909_2022_3432_MOESM1_ESM.zip › 1-Fig S3.docx]

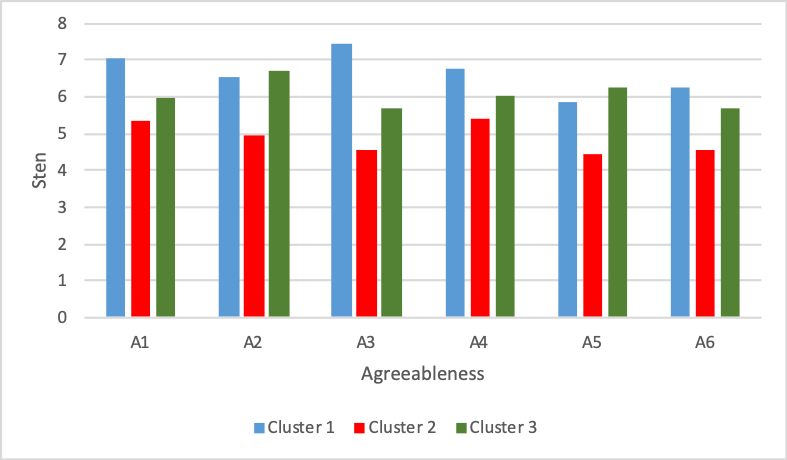


Figure S4. Mean sten values in individual clusters of the Agreeableness subdimensions

Supplement: Supplementary file 1 — Additional file 1: Figure S1. Mean sten values in individual clusters of the Neuroticism subdimensions. Figure S2. Mean sten values in individual clusters of the Extraversion subdimensions. Figure S3. Mean sten values in individual clusters of the Openness subdimensions. Figure S4. Mean sten values in individual clusters of the Agreeableness subdimensions. Figure S5. Mean sten values in individual clusters of the the Conscientiousness subdimensions. [file 12909_2022_3432_MOESM1_ESM.zip › 1-Fig S4.docx]

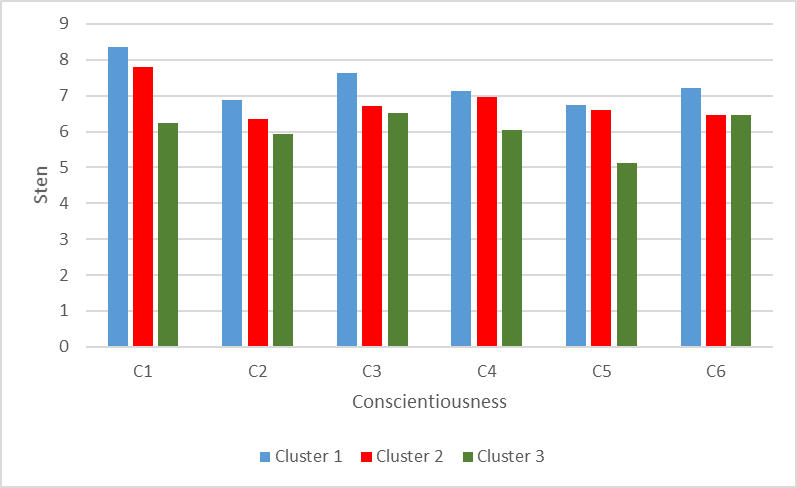


Figure S5. Mean sten values in individual clusters of the the Conscientiousness subdimensions.

Supplement: Supplementary file 1 — Additional file 1: Figure S1. Mean sten values in individual clusters of the Neuroticism subdimensions. Figure S2. Mean sten values in individual clusters of the Extraversion subdimensions. Figure S3. Mean sten values in individual clusters of the Openness subdimensions. Figure S4. Mean sten values in individual clusters of the Agreeableness subdimensions. Figure S5. Mean sten values in individual clusters of the the Conscientiousness subdimensions. [file 12909_2022_3432_MOESM1_ESM.zip › 1-Fig S5.docx]
